# Efficacy of pregabalin in the treatment of acute cerebral hemorrhage and its influence on prognosis

**DOI:** 10.3389/fphar.2026.1774707

**Published:** 2026-03-31

**Authors:** Jingchen Li, Shiqi Zhang, Youzhi Ge, Bo Sun, Chen Li, Yunpeng Shi, Xiaopeng Liu, Yuanyu Wang

**Affiliations:** Department of Neurosurgery, The Second Hospital of Hebei Medical University, Shijiazhuang, Hebei, China

**Keywords:** acute cerebral hemorrhage, NMDA, pregabalin, prognosis, α2δ1

## Abstract

**Introduction:**

Acute cerebral hemorrhage leads to high disability and mortality. Pregabalin, an α2δ1 subunit inhibitor, may reduce NMDA receptor–mediated neuronal injury and improve neurological outcomes.

**Methods:**

In this study, 158 patients with acute cerebral hemorrhage underwent minimally invasive hematoma evacuation and were assigned to receive either pregabalin or placebo for 2 weeks. Primary outcomes included hematoma volume, serum MMP2/MMP9 levels, neurological function (NIHSS), quality of life (ADL), and prognosis (GOS).

**Results:**

After 2 weeks of treatment, the pregabalin group showed significantly smaller hematoma volume compared with the control group (p < 0.01). Serum MMP2 and MMP9 levels were also significantly lower in the pregabalin group (both p < 0.01). At 12-week follow-up, patients receiving pregabalin demonstrated better neurological recovery (NIHSS, p < 0.01), improved quality of life (ADL, p < 0.01), and a higher proportion of favorable GOS outcomes (54.9% vs. 31.9%, p = 0.022).

**Conclusion:**

Pregabalin adjunct therapy significantly reduces hematoma volume, decreases MMP2/MMP9 levels, and improves neurological function and prognosis in patients with acute cerebral hemorrhage.

## Introduction

Intracerebral hemorrhage is one of the most devastating cerebrovascular diseases, characterized by extremely high mortality and disability rates, with many patients dying within the first year and the majority of survivors experiencing significant long-term functional impairment (Wang et al., 2022). The incidence is still rapidly increasing in recent years, seriously threatening human health and significantly increasing the social and economic burden.

Current treatment methods for cerebral hemorrhage include conservative treatment, craniotomy, endoscopic hematoma removal, hematoma drainage, and others (Hostettler et al., 2019). The focus of surgical treatment mainly centers on hematoma removal or drainage to relieve the compression effect of hematoma on brain tissue. Acute medical interventions targeting edema, inflammation, hemoglobin toxicity, and hematoma expansion also improve outcomes (Morotti and Goldstein, 2016). However, these approaches primarily address the mechanical consequences of bleeding and do not sufficiently target the complex secondary brain injury cascade, including excitotoxicity, oxidative stress, blood–brain barrier disruption, and neuronal apoptosis. This remains a major limitation in current clinical management.

A preliminary study revealed a simultaneous increase in the expression of the voltage-gated calcium channel protein α2δ1 subunit and the NR2B subunit of the N-methyl-D-aspartate (NMDA) receptor protein in brain ischemic tissue of experimental animals (Luo et al., 2018). A strong interaction between the α2δ1 subunit and the NR2B subunit was confirmed by immunoprecipitation (Chen et al., 2019). Targeted inhibition of α2δ1 activity with a specifically synthesized peptide significantly reduced NMDA receptor activity and improved neurological recovery in experimental animals, with no clear toxic side effects observed (Chen et al., 2019). The α2δ1 subunit is involved in the pathophysiological process of brain injury in rats with cerebral ischemia (Luo et al., 2018). After cerebral hemorrhage due to hematoma compression and surrounding secondary edema, the brain tissue around the hematoma experiences local ischemia. The site of action of the widely used clinical analgesics gabapentin and pregabalin is the α2δ1 subunit (Alles and Smith, 2018). Recent preclinical studies suggest that inhibition of the α2δ1 subunit may offer a more targeted strategy to modulate NMDA receptor–mediated excitotoxicity without the adverse effects associated with broad-spectrum NMDA antagonists. Despite this promising mechanistic rationale, there is a notable lack of clinical evidence evaluating α2δ1 inhibitors—including pregabalin—in patients with acute cerebral hemorrhage. This represents an important gap in the current literature.

Therefore, gabapentin and pregabalin, or similar drugs, have the potential to become a completely new class of drugs for cerebral hemorrhage treatment. To address this knowledge gap and provide clinically relevant data, this study investigated the effect of pregabalin as an adjunct therapy in patients with acute cerebral hemorrhage.

## Methods

### Study design

This study was designed as a randomized, controlled clinical trial conducted at the Second Hospital of Hebei Medical University between January 2020 and January 2023. A total of 158 eligible patients with acute cerebral hemorrhage were randomly assigned in a 1:1 ratio to either the observation group (pregabalin) or the control group (placebo). Randomization was performed using a computer-generated sequence, and surgeons were blinded to group allocation. All patients underwent the same minimally invasive hematoma evacuation procedure as part of the standard treatment protocol. The primary outcomes of this study were hematoma volume, serum MMP2 and MMP9 levels, and neurological function assessed by the NIHSS. Secondary outcomes included quality of life measured by the ADL scale and clinical prognosis evaluated using the GOS at 12-week follow-up. Sample size was calculated based on a pilot study expecting a 20% improvement in favorable GOS, with 80% power and α = 0.05, requiring 70 patients per group.

### Participants

Inclusion criteria: hematoma volume (calculated by Tada’s formula: height × width × length × π/6) ≤ 30 mL on the first CT examination after admission; Glasgow Coma Scale (GCS) score ≥11; onset to admission less than 12 h and onset to surgery greater than 6 h; motor dysfunction caused by cerebral hemorrhage; no vascular disease confirmed by cranial computed tomography angiography (CTA). All patients underwent emergency minimally invasive intervention to clear intracranial hematomas. All patients underwent the same surgical procedures.

Exclusion criteria: Patients with severe coagulation disorders and long-term anticoagulant use; patients with severe cardiac, renal or pulmonary insufficiency; patients with dilated pupils on one or both sides; patients with hematoma due to cerebrovascular amyloidosis or other causes; patients with disabilities or diseases that seriously affect the ability to live and work.

### Treatment

Eligible patients were randomly divided into the control group and the observation group. All patients received standard care, including emergency minimally invasive hematoma evacuation, postoperative hematoma cavity flushing, and routine medical management according to institutional guidelines. Group allocation was concealed using sealed opaque envelopes prepared by an independent research coordinator. Pregabalin and placebo capsules were identical in appearance and were dispensed by the hospital pharmacy. Surgeons performing the minimally invasive hematoma evacuation had no access to treatment assignment and were therefore blinded to patient grouping. Patients in the observation group were treated with conventional treatment and pregabalin and those in the control group were treated with conventional treatment and a matched placebo. Surgeons were blinded to patient grouping.

After the patient were bled 6 h ago, emergency minimally invasive surgery was performed to remove the intracranial hematoma. 3D CT scan was performed to determine the intracranial hematoma, a stereotactic instrument (model LEKSELL-G) was installed, lidocaine was used for local anesthesia, and the puncture target was determined by computerized 3D positioning. After routine scalp disinfection, the liquid portion of the intracranial hematoma was slowly aspirated using a drainage tube and YL-1 puncture needle (3 mm in diameter, Beijing Wantefu Technology). Subsequently, the semi-solid part of the intracranial hematoma was flushed several times with 4 °C hematoma flushing solution (epinephrine + dexamethasone + heparin). Then 2 mL of hematoma liquefying agent (urokinase + hyaluronic acid) was injected into the hematoma cavity, and the drainage tube was opened after 4 h of clamping. Postoperatively, the hematoma cavity was flushed 2–3 times per day, and the needle was removed after more than 80% of the hematoma had been cleared.

LYRICA® (pregabalin) (Pfizer Inc.) was administered orally twice daily at a starting dose of 150 mg/day and then adjusted in increments every 3 days until reaching a maximum dose of 300 mg/day. The treatment lasted for 2 weeks.

### National institutes of health stroke scale (NIHSS)

Neurological function was evaluated by the NIHSS. The NIHSS score ranges from 0 to 42. 21–42: extremely severe impairment; 16–20: severe impairment; 5–15: moderate impairment; 2–4: mild impairment; 0–1: normal status.

### Quality of life

The quality of life was evaluated by the Activities of Daily Living (ADL) scale. The ADL score ranges from 0 to 100. 60–100: basic self-care ability; 40–60: requiring help; 20–40: requiring maximum assistance; 0–20: severe dysfunction and complete life dependency.

### Glasgow outcome scale (GOS)

Clinical outcome was analyzed using the GOS. Prognosis good: daily life is not affected, symptoms and signs are mild, and patients can socialize, live and work normally; moderate disability: partial loss of ability to live and work, has neural palsy, ataxia and other disabilities but can move autonomously; severe disability: some consciousness but no independent living ability, accompanied by language and sensory impairment; plant survival: no consciousness, only exist blinking, breathing and other local movements.

### ELISA

4 mL of fasting venous blood was collected and processed by centrifugation at 2500 r/min for 15 min. The supernatant was taken and stored at −80 °C for examination. The levels of matrix metalloproteinase 2 (MMP2) and MMP9 were evaluated using corresponding ELISA kits (Abcam, Cambridge, MA).

### Statistical analysis

The statistical analysis was performed by SPSS 16.0. Data were expressed as n (percentage, %) or mean ± standard deviation (SD). Mann–Whitney test, Chi-square test, Fisher’s exact test, and repeated measures two-way ANOVA followed by Tukey’s multiple comparisons test were used as statistical tests. *p < 0.05, **p < 0.01, ***p < 0.001 and ns means no significance.

## Results

**FIGURE 1:**
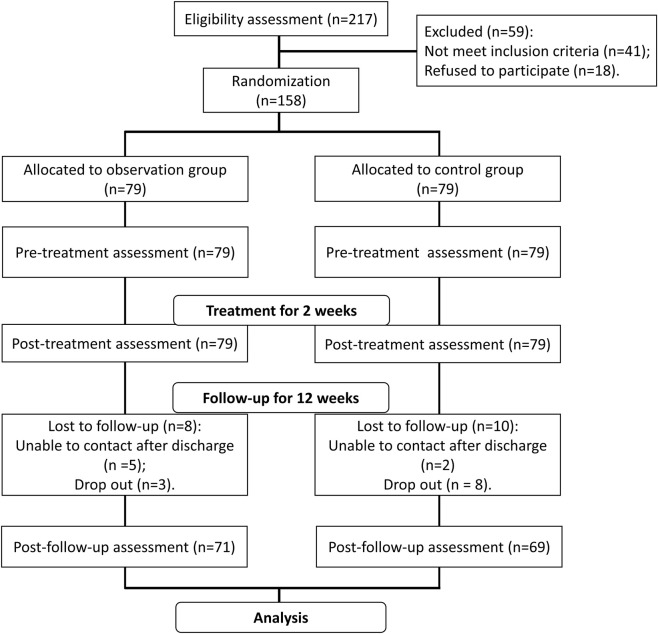
Research framework of this study.

The research framework of this study is shown in Figure 1. A total of 217 patients with acute cerebral hemorrhage were recruited, and 59 of them were excluded. Forty-one of them did not meet inclusion criteria and 18 of them refused to participate. The remaining 158 patients were randomized into the observation group (n = 79) and the control group (n = 79). After 2 weeks of treatment and 12 weeks of follow-up, 16 patients in the observation group and 20 patients in the control group withdrew.

Table 1 shows the demographic and clinical characteristics. Participants in the two groups had no significant differences in age, gender, hemorrhage location, smoking history, education level, diabetes history, hypertension history, and coronary heart disease.

**TABLE 1:** Demographic and clinical characteristics of the cerebral hemorrhage study participants.

Characteristics	Study group		p value
	Control (n = 69)	Observation (n = 71)	
Age (years)			
≤60	27 (39.1%)	35 (49.3%)	0.239
>60	42 (60.9%)	36 (50.7%)	
Gender			
Male	39 (56.5%)	38 (53.5%)	0.737
Female	30 (43.5%)	33 (46.5%)	
Time from onset to admission (h)	5.8 ± 2.1	6.6 ± 1.7	0.241
Hemorrhage location			
Thalamus	9 (13.1%)	8 (11.3%)	0.889
Lobe	13 (18.8%)	12 (16.9%)	
Basal ganglia region	47 (68.1%)	51 (71.8%)	
Smoking history			
Yes	31 (44.9%)	39 (54.9%)	0.237
No	38 (55.1%)	32 (45.1%)	
Education level			
High school and below	36 (52.2%)	42 (59.2%)	0.406
College and above	33 (47.8%)	29 (40.8%)	
Diabetes history			
Yes	23 (33.3%)	20 (28.2%)	0.508
No	46 (66.7%)	51 (71.8%)	
Hypertension history			
Yes	45 (65.2%)	52 (73.2%)	0.304
No	24 (34.8%)	19 (26.8%)	
Coronary heart disease			
Yes	10 (14.5%)	8 (11.3%)	0.569
No	59 (85.5%)	63 (88.7%)	

Values were expressed as n (percentage, %) or mean ± SD. p values for each group were derived from Mann–Whitney test. Chi-square test or Fisher’s exact test was used for assessing distribution of observations or phenomena between different groups.

**FIGURE 2:**
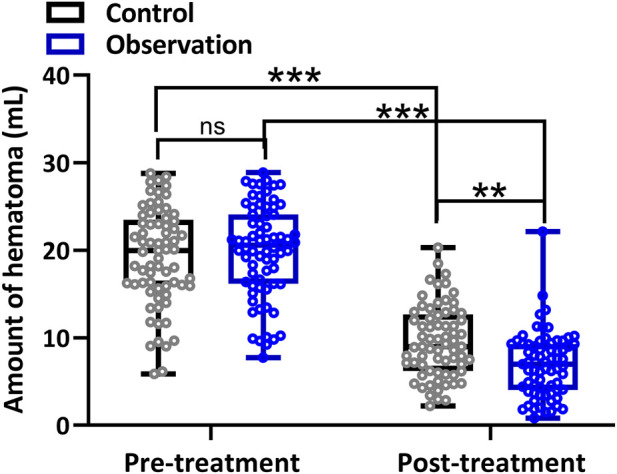
Comparison of amount of hematoma before and after 2-week treatment between the two groups. Box plot showing all the data. Repeated measures two-way ANOVA followed by Tukey’s multiple comparisons tests. **p < 0.01, ***p < 0.001 and ns means no significance.

Figure 2 shows the difference in hematoma volume. The treatment in both groups significantly reduced the hematoma volume. After 2 weeks of treatment, the observation group had a significantly smaller hematoma volume than the control group.

**FIGURE 3:**
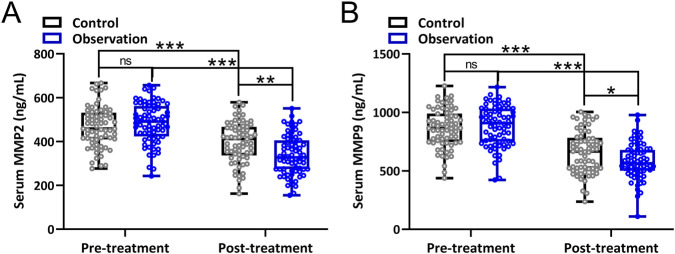
Comparisons of serum concentrations of MMP2 **(A)**, MMP9 **(B)** before and after 2-week treatment between the two groups. Box plot showing all the data. Repeated measures two-way ANOVA followed by Tukey’s multiple comparisons tests. *p < 0.05, **p < 0.01, ***p < 0.001 and ns means no significance.

MMP2 and MMP9 levels in the two groups before and after treatment are shown in Figures 3A,B. MMP2 and MMP9 are closely related to oxidative stress and brain damage. MMP2 and MMP9 declined significantly in both groups after treatment. In the observation group, MMP2 and MMP9 levels were significantly lower than in the control group after 2 weeks of treatment.

Table 2 shows the prognosis in the two groups after 3 months of follow-up using the GOS analysis. In the control group, 22 patients showed a good prognosis, 36 had moderate disability, and 11 had severe disability. In the observation group, 39 patients showed a good prognosis, 24 had moderate disability, and 8 had severe disability (Supplementary Figure S1). There was a significant difference between the two groups in the GOS prognosis analysis, with the observation group showing better prognosis after the 12-weeks follow-up.

**TABLE 2:** Comparison of the prognosis using GOS between the two groups 3 months after operation.

Characteristics	Study group		p
	Control (n = 69)	Observation (n = 71)	
Prognosis well	22 (31.9%)	39 (54.9%)	0.0225
Moderate disability	36 (52.2%)	24 (33.8%)	
Severe disability	11 (15.9%)	8 (11.3%)	
Plant survival	0 (0%)	0 (0%)	
Death	0 (0%)	0 (0%)	

Values were expressed as n (percentage, %). p value was derived from Chi-square test.

GOS: glasgow outcome scale.

**FIGURE 4:**
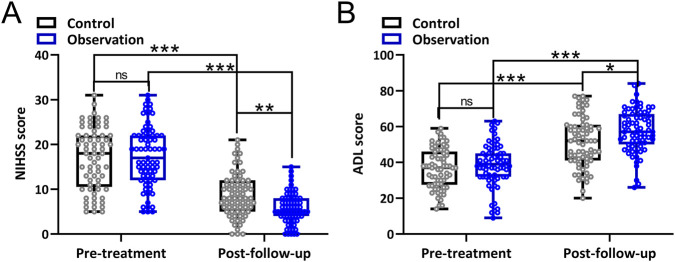
Comparisons of NIHSS **(A)** and ADL **(B)** between the two groups before and 3 months after treatment. Box plot showing all the data. Repeated measures two-way ANOVA followed by Tukey’s multiple comparisons tests. *p < 0.05, **p < 0.01, ***p < 0.001 and ns means no significance.

Neurological function of the participants was analyzed by NIHSS, and the results are presented in Figure 4A. The NIHSS score decreased significantly in both groups after treatment. After 2 weeks of treatment, the NIHSS score in the observation group was significantly lower than in the control group.

Quality of life was assessed using ADL, and the results are shown in Figure 4B. The ADL score increased significantly in both groups after treatment. After 2 weeks of treatment, the ADL score in the observation group was significantly higher than in the control group.

## Discussion

Cerebral hemorrhage is defined as bleeding caused by non-traumatic rupture of a blood vessel in the brain, which can result in blood entering the brain tissue, causing brain damage, and eventually leading to disability or death (Zhang and Ying, 2004). The specific mechanisms involved in the pathogenesis include the activation of excitatory amino acids and their receptors, persistent neuronal depolarization, the release of inflammatory factors, and the activation of protein kinases (Garg and Biller, 2019). A previous study found that among the excitatory amino acid receptors, the activation of the NMDA receptor is involved in the pathophysiology of brain injury caused by cerebral ischemia (Buchan, 1990).

Activation of NMDA receptors and the associated downstream excitotoxic signaling pathways contribute significantly to neuronal injury after intracerebral hemorrhage, as demonstrated by evidence that the α2δ-1/NMDA receptor complex mediates secondary brain damage in experimental ICH models (Li et al., 2021). Therefore, inhibiting the activation of NMDA receptors is one of the crucial mechanisms for attenuating hemorrhagic brain injury (Bullock, 1992). However, due to the wide distribution of NMDA receptors in the brain and their involvement in almost every neurological function, broad-spectrum NMDA receptor blockers can cause numerous toxic side effects and are highly constrained in clinical application (Albensi and Ilkanich, 2004). The lack of a specific drug target for NMDA receptors is a key challenge in the current research and treatment of hemorrhagic brain injury. Therefore, it is imperative to conduct in-depth research on the mechanism of neuronal damage after cerebral hemorrhage and to find new targets that can specifically reduce the activity of NMDA receptor in the peripheral damage area after hemorrhage.

The expression of the α2δ1 subunit of the voltage-gated calcium channel protein and the NR2B subunit of the NMDA receptor protein increased synchronously in cerebral ischemic tissues of experimental animals, and a strong interaction between the α2δ1 subunit and the NR2B subunit of NMDA receptor was confirmed by immunoprecipitation (Luo et al., 2018). Targeted inhibition of α2δ1 activity with a specifically synthesized peptide significantly reduced NMDA receptor activity and improved neurological recovery in experimental animals, while no clear toxic side effects were observed (Luo et al., 2018). The α2δ1 subunit is involved in the pathophysiological process of brain injury in rats with cerebral ischemia, and brain tissue around the hematoma will certainly experience local ischemia after cerebral hemorrhage due to hematoma compression and secondary edema (Luo et al., 2018). Therefore, the α2δ1 subunit may also be an important factor in brain tissue injury caused by cerebral hemorrhage, and hemorrhagic brain injury may increase the expression of the α2δ1 subunit in neurons around the hematoma, resulting in continuous activation of NMDA receptors. Inhibition of α2δ1 subunit activity or downregulation of α2δ1 subunit expression significantly reduces NMDA receptor activation and promotes neurological recovery in hemorrhagic brain injury experimental animals (Chen et al., 2018). The application of α2δ1 subunit inhibitors to reduce NMDA receptor activity may become one of the treatment strategies for cerebral hemorrhage. In our previous work, we validated the inhibitory effects of the α2δ1 inhibitor gabapentin on brain edema reduction, apoptosis inhibition, and neuroinflammation in a mouse intracerebral hemorrhage model (Li et al., 2021), leading to the protection of α2δ1 inhibitor against intracerebral hemorrhage. In this clinical study, pregabalin, as another α2δ1 inhibitor (Alles et al., 2020), was used. The current results also demonstrated the beneficial effects of pregabalin against cerebral hemorrhage in patients. Pregabalin has pharmacodynamics similar to gabapentin but is more potent in inhibiting the α2δ1 subunit (Czapinska-Ciepiela et al., 2024). Therefore, pregabalin or similar drugs may become a new class of drugs for the treatment of hemorrhagic brain injury.

The NIHSS scores were significantly lower and the ADL scores significantly higher at 3 months postoperatively in both groups compared to before treatment. The NIHSS scores at 3 months postoperatively in the observation group were significantly different from those in the control group. The GOS scores of patients in the observation group were significantly higher than those in the control group at 3 months after surgery, indicating that pregabalin treatment significantly promoted neurological recovery and prognosis. The ADL scores in both groups were significantly higher at 3 months postoperatively compared to before treatment, suggesting that pregabalin treatment could significantly improve the quality of life of patients.

MMP2 and MMP9 may exacerbate local brain tissue inflammation by degrading the vascular basement membrane, promoting neutrophil migration, damaging the blood-brain barrier, leading to brain edema and worsening of cerebral hemorrhage. Experimental results showed that elevated serum levels of MMP2 and MMP9 were significantly suppressed after pregabalin treatment and were lower than those in the control group. pregabalin may have promoted neuronal repair, accelerated neural tissue regeneration, reduced cerebral edema, and facilitated condition improvement, thereby lowering the serum MMP2 and MMP9 levels and further improving neurological function. Reduction in serum MMP2 and MMP9 levels enhances the prognosis of acute cerebral hemorrhage. Despite these promising findings, this study has several limitations. First, it was conducted at a single center with a relatively limited sample size, which may affect the generalizability of the results. Second, although pregabalin demonstrated beneficial effects on hematoma volume, MMP levels, and neurological outcomes, the follow-up period of 12 weeks may not fully capture long-term functional recovery. In addition, the GOS was the only outcome measure assessed at the 12-week follow-up because it is routinely used as the standard long-term functional evaluation tool at our institution; however, the absence of NIHSS and ADL assessments at this time point limits the comprehensiveness of long-term outcome evaluation. Third, only MMP2 and MMP9 were evaluated as biochemical markers, and additional mechanistic biomarkers could provide deeper insight into the neuroprotective pathways involved. Finally, pregabalin dosing was standardized rather than individualized, which may not reflect real-world clinical practice. These limitations should be addressed in future multicenter studies with longer follow-up and broader mechanistic assessments.

The exact mechanism by which pregabalin reduces the levels of MMPs, specifically MMP2 and MMP9, is not fully understood. However, several possible mechanisms have been proposed. 1) MMPs are a large family of calcium-dependent zinc-containing endopeptidases (Singh et al., 2015). Pregabalin can inhibit calcium influx into neurons. This reduction in intracellular calcium can decrease the activation of pathways that lead to the production and release of MMPs. 2) The binding of pregabalin to the α2δ subunit of voltage-gated calcium channels has been reported to reduce the release of several neurotransmitters, including glutamate, norepinephrine, and substance P (Gajraj, 2007). By decreasing the release of excitatory neurotransmitters like glutamate and substance P, pregabalin may reduce the activation of signaling pathways that promote MMP expression and activity. 3) Pregabalin has been shown to have anti-inflammatory properties (Abu-Rish et al., 2020). Inflammation can upregulate MMP production, so by reducing inflammation, pregabalin may indirectly reduce MMP levels. 4) Pregabalin may modulate several intracellular signaling pathways, such as the MAPK/ERK and NF-κB pathways (Verma et al., 2014), which are involved in the regulation of MMP expression. By inhibiting these pathways, pregabalin could reduce MMP production. While the exact mechanisms by which pregabalin reduces MMP2 and MMP9 levels are not fully elucidated, it is likely a combination of reduced calcium influx, decreased neurotransmitter release, anti-inflammatory effects, and modulation of intracellular signaling pathways. Further research is needed to fully understand the specific pathways involved.

## Conclusion

In conclusion, pregabalin treatment was associated with reduced hematoma volume and improved short-term neurological outcomes in patients with acute cerebral hemorrhage. However, these findings should be interpreted with caution. The study was limited by its single-center design, modest sample size, and relatively short follow-up period. Larger multicenter trials with extended follow-up are needed to validate the therapeutic potential of pregabalin before it can be considered for routine clinical use.

## Data Availability

The raw data supporting the conclusions of this article will be made available by the authors, without undue reservation.
