# Supplementary material for: Efficacy of pregabalin in the treatment of acute cerebral hemorrhage and its influence on prognosis
Source: Front Pharmacol. 2026 Mar 31;17:1774707. doi: 10.3389/fphar.2026.1774707 (PMC13077234; doi:10.3389/fphar.2026.1774707)
Supplement: Supplementary file 1 [file Image1.pdf]

Supplementary materials

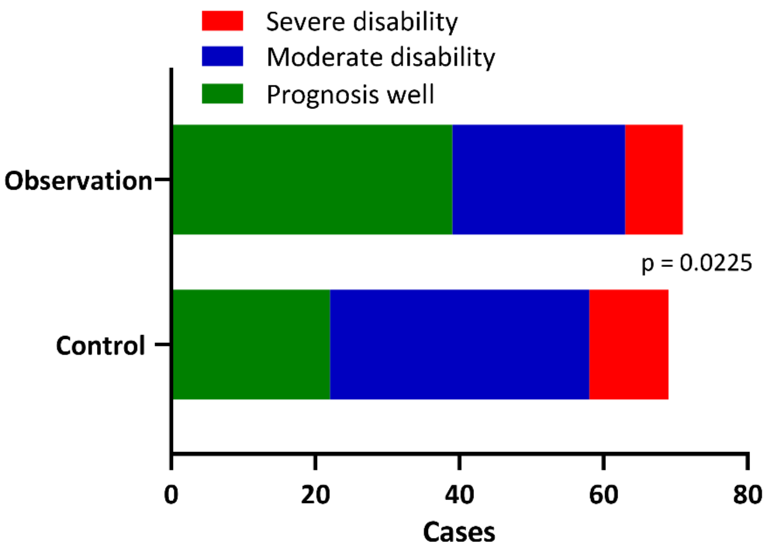

Figure S1. Comparison of the prognosis using GOS between the two groups 3 months after treatment.
